# Supplementary material for: Engineering an Effective Human SNAP-23 Cleaving Botulinum Neurotoxin A Variant
Source: Toxins (Basel). 2020 Dec 18;12(12):804. doi: 10.3390/toxins12120804 (PMC7766560; doi:10.3390/toxins12120804)
Supplement: Supplementary file 1 [file toxins-12-00804-s001.pdf]

Supplementary Materials: Engineering an effective human SNAP-23 cleaving botulinum neurotoxin A variant

Stefan Sikorra, Sarah Donald, Mark Elliott, Susan Schwede, Shu-Fen Coker, Adam P. Kupinski, Vineeta Tripathi, Keith Foster, Matthew Beard and Thomas Binz

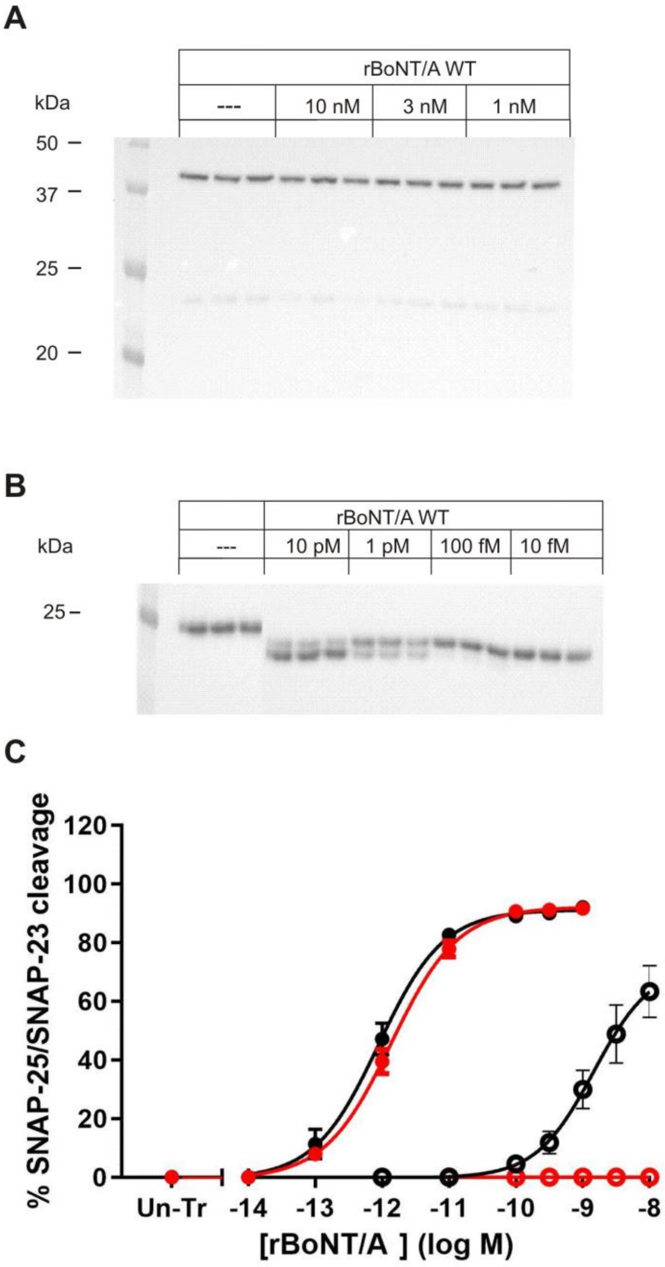

Figure S1. Activity of purified recombinant BoNT/A (E148Y, K166F, S254A, G305D) and BoNT/A wild-type in cortical neurons expressing hSNAP-23-GFP. (**A**, **B**) Three analyses conducted like those shown in Figure 2A and B were quantified. (**C**) Quantification depicted as a function of BoNT concentration. SNARE cleavage was determined for wild-type (red) and quadruple mutant (black) with SNAP-25 cleavage shown by filled circles and SNAP-23 shown by open circles. Calculated EC_{50} values are shown in Table S5.

Table S1. Amino acid sequence of LC/A mutants of the Pro-182 binding pocket obtained by yeast-based screening.

							[hSNAP-23] 20 µM		
							[LC/A] 6 µM	10 µM	% increase
							% cleavage		vs wt
	wild-type		E-148	T-307	A-308	Y-312	6.9	10.2	
mutants obtained by screening	1	GAL promoter	G	V	N	S			
	2		N	H	D	I			
	3		S	F	Y	E			
	4		M	M	T	L			
	5		H	W	V	K			
	6		I	T	I	I			
	7		M	W	D	V			
	8		H	N	N	L			
	9		E	V	N	K			
	10		L	L	S	S			
	11		I	Y	H	L			
	12		N	I	P	V	19.0	n.a.	173
	13		Y	F	N	L	14.0	n.a.	101
	14	CYC1 promoter	V	F	G	K			
	15		G	I	L	T			
	16		C	A	I	K			
	17		V	V	L	G			
	18		F	I	I	P			
	19		F	R	M	V			
	20		N	F	Y	V			
	21		Y	L	T	M	n.a.	22.8	123
	22		Y	Y	H	S			
	23		V	C	T	I			
	24		N	F	I	S			
	consensus		13/24	19/24	13/24	13/24			
		arithmetical	actual						
	hydrophobic	25	42	58	33	50	frequency (%)		
	hydrophobic	15	13	21	21	4			
	G, A, C	15	13	8	4	4			
	P	5	0	0	4	4			
	N, Q, S	15	21	4	21	17			
	acidic	10	4	0	8	4			
	basic	15	8	8	8	17			
mutants subcloned	12a		N				8.2	n.a	18
	13a		Y				57.5	n.a	728
	12b			I	P	V	12.1	n.a	74
	13b			F	N	L	10.4	n.a	49
	21 a			L	T	M	n.a	10.0	−2
	21.2		Y	L	I	M	n.a	14.1	38

Table S2. Amino acid sequence of LC/A mutants of the Lys-206 binding pocket obtained by yeast-based screening.

	wild-type		L-256	V-258	L-367	F-369	
mutants obtained by screening	1	GAL promoter	W	H	T	T	
	2		V	H	N	G	
	3		S	D	V	P	
	4		R	C	S	R	
	5		P	T	L	W	
	6		R	V	G	Y	
	7		V	A	S	A	
	8		I	A	G	W	
	9		D	S	A	S	
	10		D	A	L	G	
	11	CYC1 promoter	E	K	S	R	
	12		R	G	A	F	
	13		V	E	V	G	
	14		R	E	L	L	
	15		R	E	G	L	
	16		W	V	L	R	
	17		V	M	L	G	
	18		I	H	A	S	
	19		L	G	S	L	
	20		I	D	A	Y	
	consensus		8 + 2/20	6 + 1/20	7 + 4/20		
				5/20	7/20		
		arithmetical	actual				
	hydrophobic	25	40	15	35	20	frequency (%)
	hydrophobic	15	10	5	5	25	
	G, A, C	15	0	30	35	25	
	P	5	5	0	0	5	
	N, Q, S	15	5	5	25	10	
	acidic	10	15	25	0	0	
	basic	15	25	20	0	15	

Table S3. Activity of various LC/A mutants on SNAP-25 in standard in vitro cleavage assays.

LC/A	% Cleavage[a]	SD	No. of Experiments	Relative Activity
wild-type	60.0	10.4	20	1
E148Y	29.1	4.8	12	0.48
K166F	62.1	7.7	4	1.03
S254A	96.2	1.0	4	1.59
E148Y/G305D	37.7	2.9	4	0.62
E148Y/K166F	27.8	10.1	4	0.46
K166F/G305D	66.2	14.2	10	1.09
E148Y/K166F/G305D	60.1	3.9	8	0.99

[a] Assays were conducted as specified in Materials and Methods using a final concentration of 0.5 nM of LC/A.

Table S4. EC_{50} values for SNAP-25 and hSNAP-23 cleavage by BoNT/A quadruple (QM) and BoNT/A wild-type (WT) in cortical neurons expressing hSNAP-23-mCherry. Data are mean ± SEM of n = 4–5 independent experiments performed in triplicate. Non-parametric t-test revealed no statistical significant difference between SNAP-25 cleavage by recombinant BoNT/A wild-type and SNAP-25 cleavage by recombinant BoNT/A-quadruple mutant (p = 0.06).

	**SNAP-25**		**SNAP-25**		**hSNAP-23**	
	WT		QM		QM	
Experiment	$logEC_{50}$	pM	$logEC_{50}$	pM	$logEC_{50}$	pM
NBK68-37[a]	−11.76	1.74	−11.89	1.29	−8.11	7762.5
NBK68-240[a]	−11.82	1.51	−11.57	2.69	−9.92	120.23
NBK68-260[a]	−12.02	0.955	−11.34	4.57	−9.28	524.8
NBK83-41	−11.75	1.78	−11.75	1.78	−9.04	912.0
NBK83-111	−11.71	1.95	−11.61	2.45	−9.31	489.8
mean ± s.e. mean	−11.81 ± 0.05	1.59 ± 0.17	−11.57 ± 0.09	2.87 ± 0.60	−9.39 ± 0.19	511.7 ± 161.80

Values obtained in experiment NBK68-37 were disregarded when calculating average values. Values are outliers due to use of a different batch of Lentivirus. [a] NBK numbers refer to experiments with QM

Table S5. EC_{50} values for SNAP-25 and hSNAP-23 cleavage by BoNT/A quadruple (QM) and BoNT/A wild-type (WT) in cortical neurons expressing hSNAP-23-GFP. Data are mean ± SEM of n = 3–5 independent experiments performed in triplicate. Non-parametric t-test revealed no statistical significant difference between SNAP-25 cleavage by recombinant BoNT/A wild-type and SNAP-25 cleavage by recombinant BoNT/A-quadruple mutant (p = 0.07)

	**SNAP-25**				**hSNAP-23**	
	WT		QM		QM	
	$logEC_{50}$	pM	$logEC_{50}$	pM	$logEC_{50}$	pM
NBK83-198	−11.76	1.74	−12.1	0.79	−8.95	1120
NBK83-235	−11.82	1.51	−11.84	1.45	−8.87	1350
NBK83-258	−12.02	0.955	−12.31	0.49	−8.52	3020
NBK83-41	−11.75	1.78				
NBK83-111	−11.71	1.95				
mean ± s.e. mean	−11.81 ± 0.05	1.59 ± 0.17	−12.08 ± 0.14	0.91 ± 0.28	−8.78 ± 0.13	1830 ± 598.70
